# Mechanical and Surface-Chemical Properties of Polymer Derived Ceramic Replica Foams

**DOI:** 10.3390/ma12111870

**Published:** 2019-06-10

**Authors:** Katja Schelm, Elena Abreu Morales, Michael Scheffler

**Affiliations:** Faculty of Nonmetallic Materials, Institute for Materials and Joining Technology, Otto-von-Guericke-University Magdeburg, Große Steinernetischstraße 6, 39104 Magdeburg, Germany; elena.morales@st.ovgu.de (E.A.M.); m.scheffler@ovgu.de (M.S.)

**Keywords:** ceramic foams, polymer derived ceramics, replica technique, mechanical properties

## Abstract

Polymer derived ceramic foams were prepared with the replica method using filler free and filler loaded polysiloxane containing slurries for the impregnation of open celled polyurethane foams. A significant change in mechanical strength, porosity and surface energy, i.e., wettability after thermal treatment between 130 °C (crosslinking) and 1000 °C (pyrolysis) in argon atmosphere was observed. While low-temperature pyrolyzed foams are elastic and hydrophobic, foams pyrolyzed at high temperatures are brittle and hydrophilic, and they possess higher compression strength. Changes of these properties were correlated with the polymer-to-ceramic transformation.

## 1. Introduction

The use of polysiloxanes as preceramic polymers, also known as precursors, for the preparation of SiOC ceramics has been intensively studied over the last four decades or so. The use of preceramic polymers in ceramic processing dates back to the 1960s, when Verbeek and Yajima et al. demonstrated the use of preceramic polymers for the production of polymer-derived ceramic fibers for the first time [1,2,3]. Preceramic polymers are mainly silicon-based (low molecular weight) polymers, optional with hetero-elements included in the polymeric main chain (e.g., O in polyorganosiloxanes, C in polycarbosilanes, N in -silazanes or B in borosilanes). In dependence of the degree of functionality (amount of side groups -R) the precursors may be divided into mono- or bifunctional. The side groups -R are usually of aliphatic or aromatic nature like methyl- or phenyl-groups, or hydrogen [4,5].

Preceramic polymers can be transferred into ceramics by a defined thermal treatment including crosslinking and pyrolysis. By using preceramic polymers, it is possible to tailor a great variety of properties of the resulting polymer derived ceramics (PDCs) within wide limits, e.g., the porosity and pore structure [6], the electric conductivity [7], etc. Therefore, it is important to understand the reactions of the preceramic polymers during pyrolysis. The course from precursor to the polymer derived ceramic has been examined by many researchers analyzing the changes of typical properties such as pyrolysis weight losses, density and porosity changes and sample shrinkage [8]. The pyrolysis process was also studied by analyzing the changes in mechanical [9] and electric [7] properties as a function of conversion temperature, respectively. Ceramic microstructure, phase composition and crystallization behavior were investigated with respect to process conditions [10,11,12,13,14,15,16,17]. 

Some studies [18,19,20,21,22,23,24,25] focuses on changing the surface properties of various polyorganosiloxanes and aeorogels—in particular on the polarity that influences wetting—by changing their surface chemical functionality with increasing pyrolysis temperature. During pyrolysis between 300 °C and 700 °C, the alkyl groups facing the outside at the beginning are lost, and the more polar Si–O bonds increase in number. This means that the content of organic groups decreases from about 300 °C with increasing temperature, which makes it possible to adjust the wetting properties from wettable to non-wettable by water by choosing the pyrolysis temperature and to switch between superhydrophobic to superhydrophilic surfaces. In refs. [23,26] the preparation of cellular polymer derived ceramics via the sacrificial template method and freeze-casting of modified polysiloxanes is described. They observed a generation of additional microporosity after pyrolysis at temperatures between 500 °C and 600 °C. In this temperature range water and n-heptane vapor adsorption behavior indicated a change in the hydrophobic/ hydrophilic character, additionally. 

The replica technique is the mainly used technique for the preparation of macroporous open-cell ceramic foams with pore sizes ranging from 200 µm to 3 mm. The cellular structures are prepared by impregnating synthetic templates such as polyurethane (PU) foams or natural cellular structures with a ceramic slurry, drying the slurry and burning the template followed by a sintering process. The so prepared structures have the same morphologies as the former templates. Due to the pyrolysis of the template the ceramic struts are hollow thus degrading the mechanic strength of the cellular structures. On the other hand, those foams possess a high permeability due to their high interconnectivity of their pores. Preceramic polymers are also suitable for the replica method [27]. Ceron-Nicolat et al. [28] prepared filler loaded Si–O–C foams with a hierarchical cellular structure. Thereby the matrix foam was prepared by replication of a PU foam with the pore size of 10 pores per inch, which was further filled with a “second generation foam” with smaller pores, with a preceramic polymeric slurry containing two polysiloxanes (poly-methylphenylhydrogen-silsesquioxane and poly-methyl-silsesquioxane). These replica foams have the typical hollow struts with a triangular cross-section. Vogt et al. [29] prepared nitrogen-bonded silicon carbide foams by nitridation at 1400 °C using a slurry made of poly-methyl-silsesquioxane, SiC and Si. To fill the hollow struts and to increase the strut thickness, several re-infiltration and pre-sintering steps were performed. Nangrejo and coworkers [30,31,32,33,34] and Sorarù et al. [35] used a modified replica method to prepare open-cellular polymer derived ceramic SiOC and SiC foams with dense struts. Therefore, a PU foam was impregnated with a preceramic polymer solution, with and without fillers, followed by pyrolysis in argon or nitrogen atmosphere.

However, all published studies preparing replica foams with preceramic polymers analyzed only the properties of the pyrolyzed foams or the high-temperature behavior above 1000 °C, such as phase separation and crystallization or nitration. Takahashi et al. [36,37,38] investigated the development (change in phase morphology, composition and dimensions) during pyrolysis of foamed PU preceramic polymer mixtures by gravimetric and infrared spectroscopic measurements in the temperature range from room temperature to 1400 °C. The analysis of polymer-to-ceramic conversion of preceramic polymers has been performed in the literature on powders or compact samples to suffice. To our knowledge, the behavior during the burning out of the PU and pyrolysis and the associated changes in properties have not yet been investigated on replica foams.

In this study the transformation of reticulated polymer derived ceramic foams and the change of their properties during the polymer-to-ceramic conversion under inert gas will be discussed. A major aspect of this study is dedicated to surface-chemical properties and their changes as a function of the processing conditions; with respect to this, wettability control may lead to novel applications of PDC foams [25].

## 2. Materials and Methods 

### 2.1. Foam Preparation

Open-cellular polyurethane foams (Koepp Schaum GmbH; Oestrich-Winkel, Germany) with a pore size of 20 ppi and a dimension of 15 mm × 15 mm × 20 mm were used as templates for the replication process. The polyurethane foams were coated with different preceramic polymer slurries without particulate fillers, with Al_2_O_3_ (CT3000SG; d_50_: 0.5 µm; Information from manufacture datasheet; Almatis GmbH, Frankfurt, Germany) and with SiC as (SiC-SM07; d_50_: 1.3–1.7 µm; Information from manufacture datasheet; ESK-SIC GmbH, Frechen, Germany); SiC and Al_2_O_3_ both as inert fillers. As pre-ceramic polymers two commercially-available polysiloxanes were used: a solid poly-methyl-silsesquioxane (SILRES^®^ MK) and a liquid poly-methylphenylvinylhydrogen-silsesquioxane (SILRES^®^ H62 C), both from Wacker Silicone AG, Munich, Germany. The first step for preparing the slurry was to dissolve the siloxanes in methyl-triethoxy-silane (MTES; Sigma-Aldrich Chemie GmbH, Steinheim, Germany) by mixing with a magnetic stirrer. When fillers were used, they were added after the dissolution of the preceramic polymer in silane. Then oleic acid (GPR RECTAPUR^®^, VWR International S.A.S., Fontenay-sous-Bois, France) and aluminum acetylacetonate (Merck KGaA, Darmstadt, Germany) were added to the slurry as crosslinking catalysts for the MTES-SILRES^®^ MK-system. The SILRES^®^ H62 C contains a Pt-catalyst for crosslinking via a hydrosilylation reaction. The compositions of the slurries are shown in Table 1. The ratio of the preceramic polymers in the filler free and filler loaded slurries are slightly different; the rheological behavior of the slurries was adjusted to achieve a viscosity suitable for the coating process. In the filler free slurry, it was necessary to increase the amount of SILRES^®^ MK to increase the viscosity, so that the slurry did not drain off the struts. For the coating process the templates were dipped into the slurries so that they were completely wetted. Excess slurry was removed manually and closed windows were opened by blowing air through the foams. 

The impregnated foams were dried in air for at least 24 h before crosslinking in a circulating air furnace (KU 40/04/A, THERMCONCEPT Dr. Fischer GmbH, Bremen, Germany). Crosslinking was performed stepwise at 60 °C, 90 °C and 130 °C with holding times of 3 h, 3 h and 24 h, respectively. The heating and cooling rates between the holding steps were 2 K min^−1^. 

Pyrolysis was terminated for each set of samples at 130 °C, 300 °C, 400 °C, 500 °C, 600 °C, 800 °C and 1000 °C. The holding time was 3 h each and the heating and cooling rate were set to 1.5 K min^−1^. The pyrolysis was performed in flowing argon atmosphere with a gas flow rate of 10 l h^−1^ in a tube furnace (ROC 75/450/16, THERMCONCEPT Dr. Fischer GmbH, Bremen, Germany).

### 2.2. Characterization

The geometric dimensions of the crosslinked, and pyrolyzed foams were measured with a Vernier caliper. The volume shrinkage ($S_{V}$) of the foams after pyrolysis at different temperatures was calculated with the volumes of the foams:

(1) $$S_{V}=\frac{V_{cross-linked}-V_{pyrolyzed}}{V_{cross-linked}}.$$

The geometrical density ($\rho_{g}$) was determined as the mass-to-volume-ratio. True densities ($\rho_{t}$) of the strut material, without any pores, were determined by He-gas pycnometry (AccuPyc 1330®, Micromeritics GmbH; Aachen, Germany) of powdered and milled foams.

The total porosity (P) of the foams was calculated using the following Equation:

(2) $$P=1-\frac{\rho_{g}}{\rho t}.$$

The polymer-to-ceramic conversion of the crosslinked preceramic slurries and the pyrolysis of the PU template with respect to weight change were analyzed by thermal analysis using a Netzsch STA 449F3 Jupiter equipment (NETZSCH-Gerätebau GmbH, Selb, Germany). The measurement was performed with a heating rate of 10 K min^-1^ from room temperature to the maximum temperature of 1000 °C in flowing argon atmosphere with a gas flow rate of 50 mL min^−1^.

The cellular structure was analyzed by microcomputed tomography (µ-CT, Phoenix Nanotom S; GE Sensing & Inspection, Wunstorf, Germany), using a tungsten target operating at 60 kV and 160 µA. The distances between the sample holder, the X-ray tube and the detector were chosen so that a constant voxel size of 2 µm resulted from these adjustments. The reconstruction to the three-dimensional volume was carried out with the Phoenix Datos X 2.0 program package (GE Sensing & Inspection, Wunstorf, Germany). Calculations of the strut thickness and window diameter distribution were performed with the program package CTAnalyzer 1.17 (CTAn, Skyscan/Bruker microCT, Kontich, Belgium) using the three-dimensional reconstructed volume. Therefore the data were binarized by determining a binarization threshold value to differentiate between strut material and air using a specific manual definition of this threshold value as described in Reference [39].

Microstructure was analyzed by light microscopy (VHX-500F; KEYENCE Deutschland GmbH; Neu-Isenburg, Germany) and scanning electron microscopy (XL30 ESEM-FEG; FEI Company, Hillsboro, OR, USA). 

**Figure 1:**
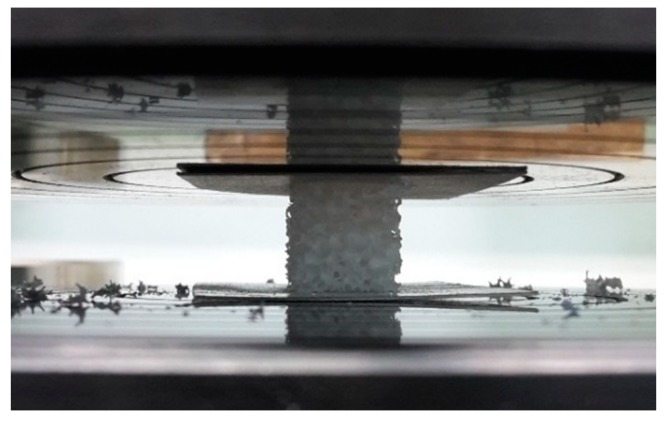
Setup of the compressive strength measurement. The cardboard placed on top and underneath the specimen is visible.

The compressive strength of the pyrolyzed and crosslinked samples, which were prepared with PU-templates with the dimensions of 15 mm × 15 mm × 20 mm, was determined using a TIRAtest 2825 testing machine with circular loading plates (diameter: 150 mm) (TIRA GmbH, Schalkau, Germany). To ensure a more homogenous load on the samples a cardboard piece with 1 mm thickness was placed between the loading plates and the sample because of sticking out struts. The applied head moving speed was set to 2 mm min^−1^. Ten samples of every foam series were measured, whereby the samples were put on the plane site. The measurement settings were selected according to the test configurations chosen in References [40,41]. A photo of the setup and a tested specimen is shown in Figure 1.

For interpretation of the results a two parameter Weibull distribution was used and the Weibull modulus m and the characteristic compressive strength σ_cr_ were calculated. According to [42] the compressive strength $\sigma_{cr}^{*}$ depends on the relative density of the foams ρ*ρs and on the bending strength of the strut material $\sigma_{b}$:

(3) $$\frac{\sigma_{cr}^{*}}{\sigma_{b}}=C{\left(\frac{\rho^{*}}{\rho_{s}}\right)}^{n}$$

The parameters C and n were set to 0.2 and 3/2 as suggested in [42] for open-cellular brittle foams. The bending strength was set as a free variable while fitting the Ashby line.

In the elastic region, the behavior during compression of ceramic foams is characterized by bending. This is described by [42] with the same type of equation as Equation (3):

(4) $$\frac{E}{E_{s}}=C_{1}{\left(\frac{\rho^{*}}{\rho_{s}}\right)}^{n}$$

This relates the foam’s effective elastic modulus E to the relative density, the elastic modulus E_s_ of the bulk material and a constant C_1_. C_1_ was set to 1 and n to 2 [42]. The linear-elastic region of the stress-strain curves were used for fitting the results and E_s_ was calculated. 

The surface of particular struts was analyzed by confocal Raman microscopy (laser wavelength: 532 nm; Alpha 300R, WITec GmbH, Ulm, Germany) measuring single spectra of samples pyrolyzed at different temperatures.

The contact angle was determined on planar tapes made of the preceramic slurry without and with fillers, which were heat treated in the same way as the foams, in order to determine the effect of the change in chemical composition during pyrolysis on the wetting behavior. Contact angles were measured with water and diiodomethane (Merck Schuchardt OHG, Hohenbrunn, Germany) as testing liquids with a contact angle meter (OCA 20, DataPhysics Instruments GmbH, Filderstadt, Germany). For calculation of the surface free energy according to the method of Owens, Wendt, Rabel & Kaelble [43] the SCA20 software was used (V. 4.2.4, DataPhysics Instruments GmbH, Filderstadt, Germany). The following values for water and diiodomethane were chosen according to the DataPhysics database; water: σ_l_: 72.8 mN m^−1^, $\sigma_{l}^{D}$: 21.8 mN m^−1^, $\sigma_{l}^{P}$: 51.0 mN m^−1^; diiodomethane: σ_l_: 50.8 mN m^−1^, $\sigma_{l}^{D}$: 50.8 mN m^−1^, $\sigma_{l}^{P}$: 0.0 mN m^−1^), whereby σ_l_, $\sigma_{l}^{D}$ and $\sigma_{l}^{P}$ stand for the surface tension, the disperse and polar content of the free surface energy of the testing liquids.

## 3. Results and Discussion

### 3.1. Microstructure and Shrinkage 

**Figure 2:**
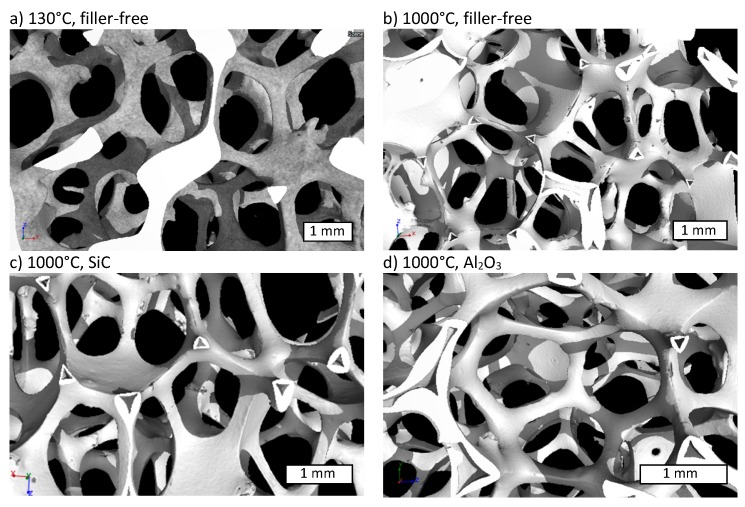
X-ray µ-CT reconstruction image of a volume element representing the typical structure of (**a**) a crosslinked filler-free foam and (**b**) of a filler-free foam pyrolyzed at 1000 °C. Foams pyrolyzed at 1000 °C with fillers are shown in (**c**) with SiC as filler and in (**d**) with Al_2_O_3_ as filler. The white bars indicate the length scale of 1 mm.

**Figure 3:**
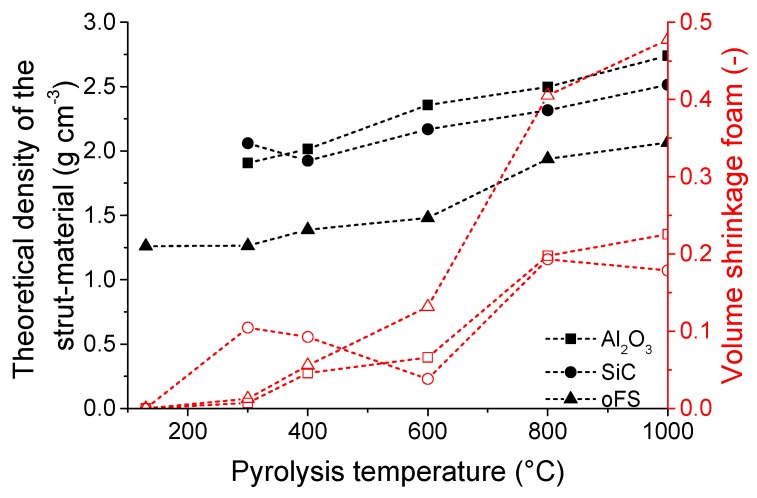
Dependence of the theoretical density of the strut material measured on crushed filler-loaded (Al_2_O_3_, SiC) and filler-free foams (oFS) and volumetric shrinkage of the temperature treated foams in dependence of the pyrolysis temperature.

**Figure 4:**
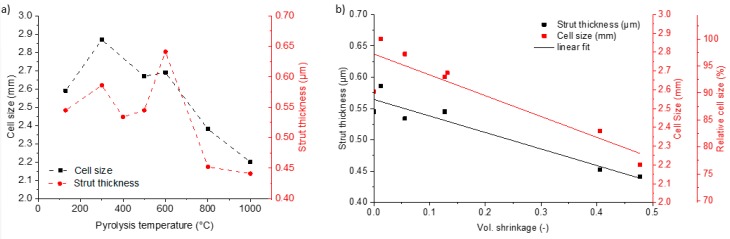
(**a**) Maxima of the strut thickness and the cell size distribution in the pyrolyzed foams in dependence of the pyrolysis temperature; (**b**) Strut thickness and cell size and relative cell size as function of the volume shrinkage of foams.

As shown in the reconstructed 3-dimensional µCT-structure, the open cellular form of the PU template is well replicated after the crosslinking with the filler free preceramic slurry at 130 °C (Figure 2a) and also after pyrolysis, e.g., at 1000 °C (Figure 2b). The struts of the crosslinked foam appear dense because of the included PU-template foam, which is not yet burned-out at the crosslinking temperature of 130 °C, whereas the pyrolyzed foams show the replica-typical hollow triangular hollow struts. In case of the pyrolyzed foam without fillers some cracks appear (see Figure 2b), running along the sharp edges of the struts. In contrast the pyrolyzed foams with fillers do not show any crack formation (see Figure 2c,d). The reason for crack formation is the large volume shrinkage of preceramic polymers during pyrolysis. At a pyrolysis temperature of 1000 °C the foams have a shrinkage of 48 vol%, whereas the filler loaded slurries caused a shrinkage of only ~20 vol% (see Figure 3). These cracks appear in the filler free samples at temperatures higher than 600 °C. Above this temperature the shrinkage rises sharply from 15 vol% at 600 °C close to 50 vol% at 1000 °C for the filler free samples and leads to the formation of the cracks. By adding inert fillers, the shrinkage was significantly reduced, and no cracks were observed in the pyrolyzed foams, even at high pyrolysis temperatures of 1000 °C. The shrinkage of the foams during pyrolysis may be comprehended by examining the development of the cell size and the strut thickness (Figure 4a). The cell size and strut thickness reach their maximum at 2.9 mm and 0.6 mm, respectively, at a pyrolysis temperature of 300 °C, respectively and decrease to 2.2 mm or 0.45 mm, respectively after pyrolysis at 1000 °C. When plotting the window size and structure thickness against the shrinkage (see Figure 4b), a nearly homogenous shrinkage is indicated; both values are linearly fittable. The correlation coefficient for both fits were 0.82. The slope of the fitted curve for the relative cell size is −1.05 mm, that means the shrinkage of the cell size corresponds approximately to the theoretical value of −1.16 mm. For calculation of the theoretical value the cell was assumed as a sphere and the diameter d was assumed as cell size. During shrinkage the volume of the sphere is reduced and the dependency of the diameter to the volume is $\pi d^{3}/6$. The reduction in diameter was related to the reduction in sphere volume corresponding to the volume shrinkage. The slope of the development of the strut thickness is –0.26 µm, what is in good accordance with the theoretical shrinkage (−0.22 µm) of the strut radius, due to the dependence of the radius to the volume with the third root. 

The addition of approx. 20 vol% SiC or Al_2_O_3_ as fillers leads to a reduction in the shrinkage, so that the maximal shrinkage of these foams equals to ~20 vol% after pyrolysis at 1000 °C. The volume shrinkage is caused by the density change during polymer-to-ceramic transformation, resulting in a significantly denser strut material after pyrolysis. Due to the addition of fillers the theoretical density of the foams’ strut material (as measured on crushed foams) is higher than that without fillers. The density of the crosslinked filler-free precursor-containing coatings on PU sponge templates is 1.26 g cm^−1^ and increases during pyrolysis up to 1000 °C to 2.07 g cm^−1^. The density of the strut material with fillers increases from 2.06 g cm^−1^ to 2.71 g cm^−1^ for SiC-filler and from 1.91 g cm^−1^ to 2.74 g cm^−1^ for Al_2_O_3_-filler. All foams have high total porosities of 90 % to 96 %, whereby the filler-free samples have the lowest total porosity values, due to higher shrinkage. 

**Figure 5:**
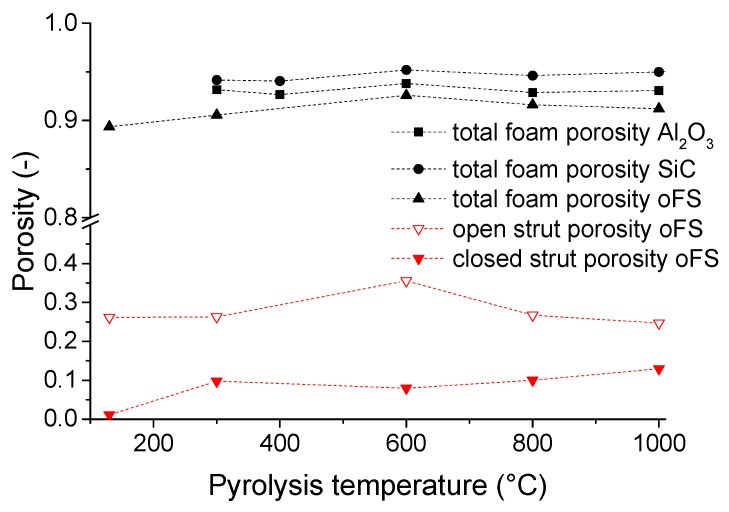
Geometrical foam porosity from the filler-free and filler-loaded samples and open and closed strut porosity of the filler-free samples measured with the Archimedes principle in dependence of the pyrolysis temperature.

During pyrolysis, a specific amount of transient porosity was present in the preceramic material due to the decomposition of the polymer and gas evolution. Figure 5 shows the open and closed porosity of the filler-free crosslinked and pyrolyzed foams, respectively, measured with the Archimedes’ method (according to the DIN EN 623-2:1993-11 standard [44]) using ethanol as solvent. The struts show a relatively high total strut porosity between 30 % and 45 %, whereby the hollow struts of the foams are also accounted as strut porosity. As the PU template is still present in the crosslinked, not yet pyrolyzed foams and residues of the template are present in the foams pyrolyzed at 300 °C, the open porosity of the struts is still low. The foams pyrolyzed at 600 °C show the highest open strut porosity. In this temperature region (400 °C–800 °C) the main mass loss of the preceramic polymers SILRES® MK and H62 C occurs (see Section 3.2.1) due to splitting of organic groups (compare Raman measurements in Section 3.2.2). The mass loss, accompanied by the release of the gaseous by-products build the open porosity. With increasing the pyrolysis temperature, the open porosity is transformed into closed porosity. At this temperature the foams also have the highest total porosity as the shrinkage has not yet started to a major extent (shrinkage “oFS” ~15 %) but the weight loss of the foam (PU-template and coating material (“oFS”)) is already 60 %. This development of the porosity is in accordance to other experiments. Schmidt et al. [45,46] analyzed polymer derived ceramic bulk and foamed samples regarding the porosity. At a pyrolysis temperature of 600 °C a maximum in porosity and specific surface area was achieved. At temperatures >800 °C, the main porosity got lost. The development of the amount of porosity can be controlled by the addition of solid filler particles, which hindered the elimination of porosity due to the limitation of shrinkage. 

### 3.2. Compositional and Structural Change 

#### 3.2.1. Thermal Analysis

**Figure 6:**
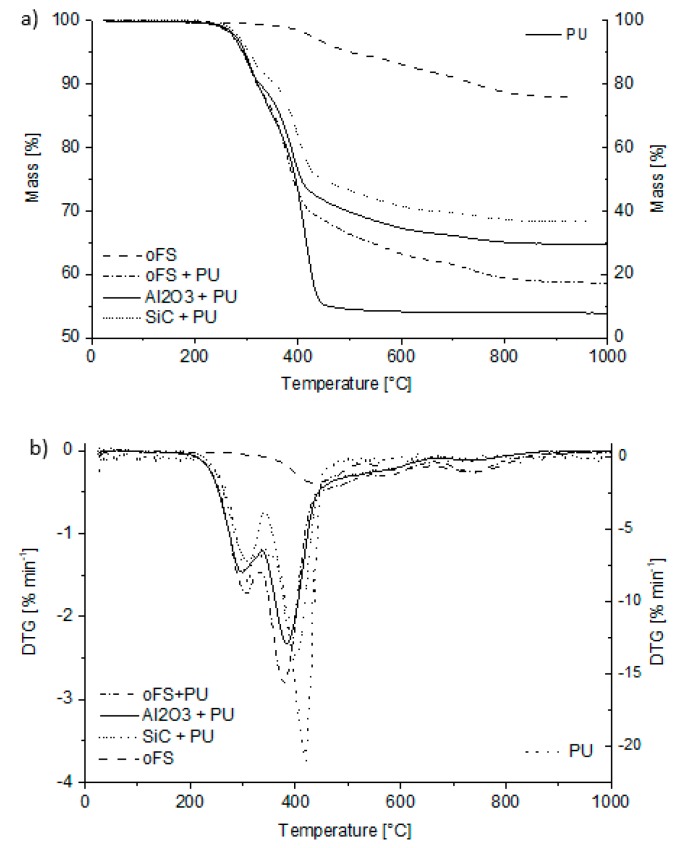
(**a**) Thermogravimetric analysis of the preceramic coating material without fillers (oFS), of the polyurethane foam template (PU) and of the crosslinked coated foams with (Al_2_O_3_ + PU, SiC + PU) and without fillers (oFS + PU) including the polyurethane template; (**b**) Differential thermogravimetric (DTG)-signal of the thermogravimetric signals.

Parallel to the increase in density, the preceramic polymers showed specific mass losses during heating. These transformation reactions were studied by thermal gravimetric analysis (TGA) and Raman microscopy. The results of the thermogravimetric analysis are shown in Figure 6. The crosslinked coating materials with and without filler show different steps in mass loss. These steps can be correlated to specific reactions that occur during the pyrolytic polymer-to-ceramic transformation in inert gas of crosslinked poly-organosiloxanes with methyl-, ethyl-, phenyl- or ethoxy-side groups. According to the literature these reactions are listed below [7,8,9,10,11,12,13,14,15,16,17]:

1. < 400 °C: Formation and release of water and ethanol due to remained crosslinking active groups like Si–OH and/or Si–OC_2_H_5_
2. ~550–1000 °C: Redistribution reactions involving the exchange of Si-O, Si-H and Si-C bonds with the release of volatile silicon compounds (e.g., Me_3_SiOSiMe_3_);
  ~600–700 °C: Possible release of small amounts of higher linear or cyclic polysiloxanes and/or tetramethylsilanes
3. Radical reactions and release of gaseous products with formation of an open porous network.
  In dependence of the bonded groups the following temperature steps are ascertainable:
  ~300–700 °C: Cleavage of Si–(C_6_H_5_) bonds and release of benzene (for PhSiO_1.5_)
  ~600–900 °C: Cleavage of Si–(CH_3_) bonds and release of methane (for MeSiO_1.5_)
  ~600–1100 °C: Dehydrogenation
4. > 800 °C: Formation of aromatic carbon/ free carbon
5. > 1000 °C–1600 °C: Enrichment of SiO_4_- and SiC_4_-units and precipitation of nanocrystals of SiO_2_ and SiC due to further chemical bond redistribution
6. > 1200 °C–1500 °C: Carbothermal reduction of SiO_2_ and C according to the following reaction equations [16]:
  SiO_2_ + 3 C → SiC + 3 CO
  If y > 1 + x: SiO_x_C_y_ → SiC + x CO + (y − x − 1) C
  If y < 1 + x: SiO_x_C_y_ → [(x + y − 1)/2] SiC + [(x + y −1)/2 CO + (x – y + 1) SiC
7. > 1550 °C: Graphitization/ crystallization of the carbon and crystallization of SiC

There is only a small weight loss step seen at crosslinking temperatures up to 300 °C, because the samples measured in Simultaneous Thermal Analysis (STA) were already crosslinked at 130 °C. The higher weight loss step at 400 °C–500 °C is assigned to the loss of silicon containing oligomers. At temperatures higher than 600 °C there are two further weight loss steps resulting from the split off of the phenyl- (maximum rate at ~600 °C) and methyl-groups (maximum rate at ~750 °C) from the SILRES® H62 C and SILRES® MK resin, respectively. These results are in good accordance with the literature as described above [9]. The pyrolytic gases consist mainly of benzene or methane, respectively [12]. The ceramic yield of the filler-free preceramic coating system was ~88 wt%. 

**Figure 7:**
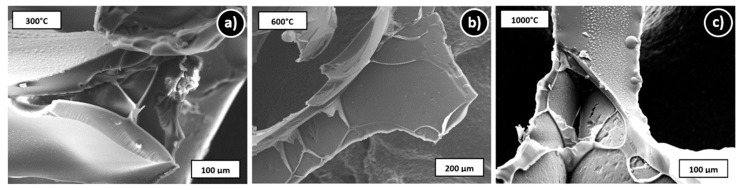
SEM images of hollow struts of filler-free foams at different degrees of pyrolysis; (**a**) 300 °C, (**b**) 600 °C, (**c**) 1000 °C.

**Figure 8:**
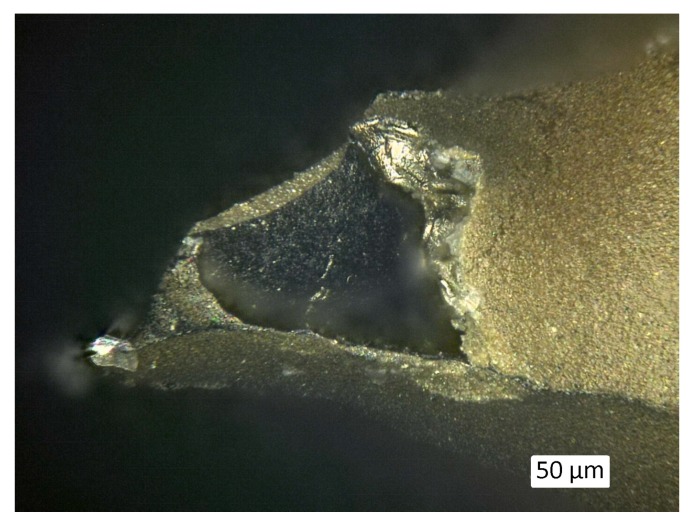
Light microscope image of a SiC-filled foam after pyrolysis at 600 °C in argon atmosphere.

Figure 6 also shows the results of the thermogravimetric analysis of the polyurethane foam in argon atmosphere. The degradation of the PU takes place in a temperature range from 250 °C to 450 °C. A foamed polyether polyurethane was thermally analyzed by Takahashi et al. [37] using the thermogravimetric analysis and Fourier transformed infrared (TGA-FTIR) method under nitrogen. Two degradation steps were also observed between 300 °C and 500 °C. At the first step at 325 °C the urethane linkages degrade and low molecular weight components (mainly aliphatic fragments) are released. The second degradation step occurs at 400 °C, whereby oligomeric isocyanate and high-molecular weight polyether polyols are released. Because of the heating in argon pyrolysis and PU degradation is not complete and a residue of ~8 wt% remains after being heated to 1000 °C (see Figure 6). Thermal analysis of the PU foams in air indicates a complete decomposition at 600 °C (results not shown here). In SEM images (Figure 7) thin walls on the surface of the hollow struts are visible after each pyrolysis temperature above 300 °C. EDS measurements detect mainly carbon (results not shown here). But the PU residue is also present on the outer foams’ surface as indicated by Raman measurements (see Section 3.2.2), although they are not visible under the light microscope (Figure 8), where only black discoloration in the inner of the hollow strut is visible. 

The thermogravimetric analysis results and the DTG-signals of a coated and crosslinked foam are shown in Figure 6, and they represent a combination of the TGA results from the pure polymeric coating material and the PU foam. The ceramic yield of the pure polysiloxanes is 83.5 wt% for SILRES® MK and 76.1 wt% for SILRES® H62 C after pyrolysis in argon atmosphere.

The addition of 18.8 vol% Al_2_O_3_ or 22,4 vol% SiC (~46 wt%) in the slurry reduces the mass loss of 42 wt% to 22 wt% or 26 wt%, respectively. The slightly different mass losses of the filled foam samples (Al_2_O_3_ + PU and SiC + PU) are a result of probably some inhomogeneties regarding the coating thickness on foam samples and thus a slightly different PU content. 

#### 3.2.2. Raman Microscopy

Raman spectra were recorded to observe the changes in chemical bonds, and thus, in the composition of the precursor during thermal treatment. The results of the Raman microscopy measurements of the crosslinked and of the pyrolyzed foams are generally in good agreement with the results of thermogravimetric measurements. To obtain information of the influence of the template on the resulting foam composition Raman measurement were performed on the outer surface of foams and also on the inner surface of the struts.

**Figure 9:**
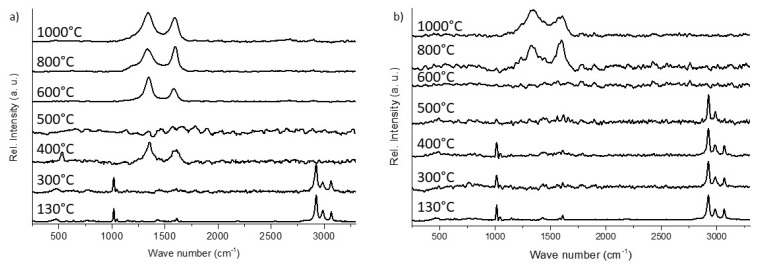
Raman microscopic analysis of the surface of crosslinked and pyrolyzed foams after pyrolysis at different temperatures. (**a**) Raman spectra of foams embedded, ground and polished as measured on the inner of the foam struts; (**b**) Raman spectra of the same foams, where the measurement position was on the outer surface of the foam struts.

The Raman spectra of the inner strut material of crosslinked preceramic foams (Figure 9a) show typical peaks for foams containing organic groups (–C_6_H_5_, –CH_3_, –C_2_H_3_ for SILRES® H62 C and –CH_3_, –OC_2_H_5_ for SILRES® MK): Si–CH_3_ vibration at 2921 cm^−1^ (≡Si–CH_3_ symmetric vibration) and 2982 cm^−1^ (≡Si–CH_3_ asymmetric vibration) as well as Si-C_6_H_5_ vibration at 1011 cm^−1^ and 3062 cm^−1^ (C–H stretching vibrations). In the literature less information is found, where the polymer-to-ceramic transformation is analyzed with Raman spectroscopy; however, other methods, like mass spectrometry [8,13,14,15,17], ^29^Si and ^13^C nuclear magnetic resonance [10,15,16] and Fourier transform infrared spectroscopy [8,9,17,36,47], were used to analyze the thermal transformation with respect to the chemical bonds and their changes as a function of temperature. These results are in good agreement with the above cited literature. The crosslinked material shows a spectrum, in which the peaks belonging to the methyl and phenyl groups are visible up to a pyrolysis temperature of 400 °C. At a pyrolysis temperature of 500 °C the peaks belonging to phenyl groups, which are present in the SILRES®H62 C, disappear and the signals assigned to methyl groups, as present in both polysiloxanes -SILRES®H62 C and SILRES®MK- were detected. The split off of these two functional groups can also be seen in the two weight loss steps in the STA-measurement shown in Figure 6b. Phenyl groups are released at lower temperatures during pyrolysis due to the weaker Si–phenyl bonding strength compared to the bond strength between Si and methyl groups (dissociation energies for Si–C: Si-methyl: 318 kJ mol^−1^, Si-phenyl: 215 kJ mol^−1^) [9]. Foams pyrolyzed at temperatures higher than 600 °C show that the two peaks originate from free carbon (D-band: 1350 cm^−1^, G-band: 1582 cm^−1^) that is formed during pyrolysis. 

The comparison of Raman spectra of foams where the outer surface was measured (Figure 9b) shows signals assigned to free carbon already after pyrolysis at 400 °C as the lowest pyrolysis temperature. These carbon species originate from an incomplete decomposition of the PU template and part of these residuals deposit on the outer surface of the foams. Also, on the inside of the hollow struts, some residuals are visible, as shown in Figure 7 and Figure 8.

#### 3.2.3. Wetting Behavior as a Function of Pyrolysis Temperature 

**Figure 10:**
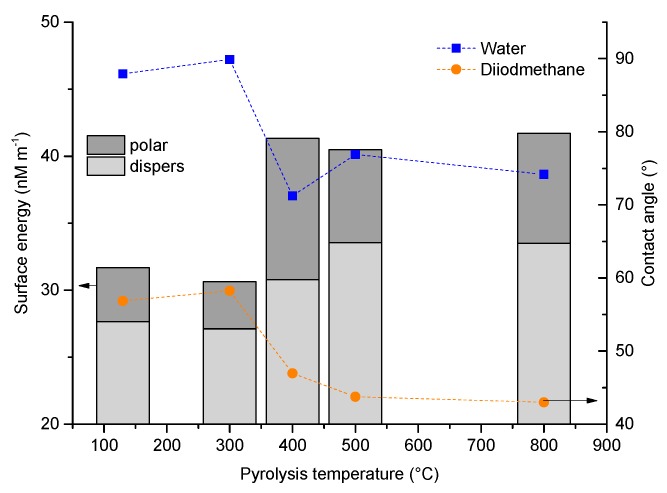
Contact angle of water and diiodomethane and the free surface energy as a function of the pyrolysis temperature of the PDC material.

Figure 10 shows the surface free energy with its polar and its disperse part of crosslinked and pyrolyzed foams, determined with the OWRK method [43] according to Owens and Wendt, Rabel and Kaelble; the contact angles of water on PDC tapes and of diiodomethane and PDC tapes were measured. The planar tapes possess the same composition as the foams. Water and diiodomethane droplets were placed on the surface of the preceramic and PDC tapes; measuring contact angles on the small and curved struts was not possible. The contact angles of both, water and diiodomethane, decrease with increasing pyrolysis temperature of the tapes and the surface energy increases. The reason for the increase in the resulting wettability with increasing pyrolysis temperature is the split off of the organic groups bonded to the siloxane backbone, which means that the hydrophilic character of the system increases; see also [25] for PDC coatings on Al_2_O_3_.The loss of organic groups is confirmed by Raman measurements (Figure 9) and the weight loss during thermal analysis (Figure 6). The release of the organic groups starts between 300 °C and 400 °C; in this temperature region there is also a step in the contact angle curve spanning from 90° to 75° and from 60° to 43° for water and diiodomethane, respectively. This results in a change from a hydrophobic (contact angle θ with water 90° < θ < 150° [48]) to a hydrophilic (0° < θ < 90° [48]) wetting behavior. Parallel to a decrease of the contact angles values, the free surface energy increases within this temperature region, and no differences between pyrolysis in argon or nitrogen were observed up to a pyrolysis temperature of 1000 °C. The change of the contact angle with increasing pyrolysis temperature is less distinct within this set of tape samples as in comparison to prepared coatings of a comparable slurry [25]. The larger range of achieved contact angles is probably based on a different surface roughness. There contact angles between 110° and 20° were achieved when using a filled preceramic slurry. 

#### 3.2.4. Compressive Strength as a Function of Pyrolysis Temperature

Determination of the characteristic compressive strength and the Weibull modulus were used to study the effect of the pyrolysis temperature on the mechanical properties of the polymer derived foams. 

**Figure 11:**
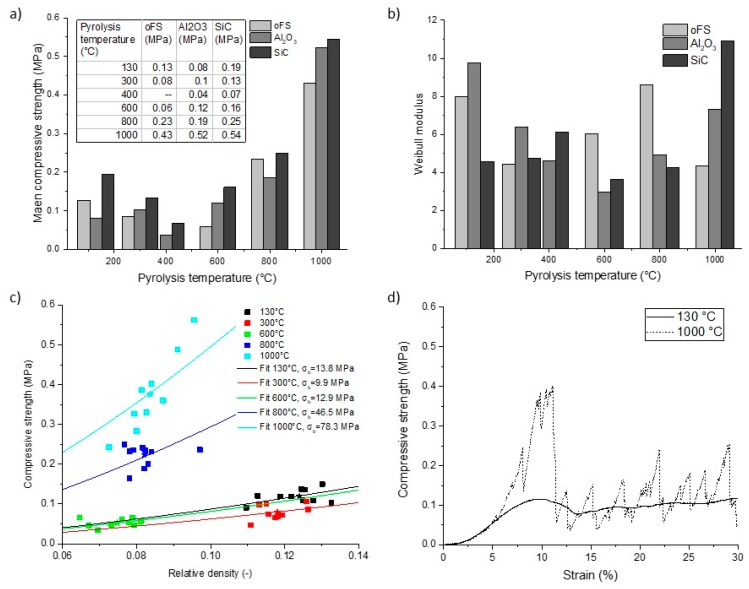
(**a**) Compressive strength of foams with fillers and of the filler-free foams as a function of the crosslinking / pyrolysis temperature. (**b**): Weibull modulus of the foams pyrolyzed at different temperatures. (**c**) Compressive strength of pyrolyzed foams against relative density including the fits according to Equation (3) with n = 3/2 and C = 0.2. The fitted bending strength σ_b_ is given in the legend. ★ indicates the corresponding median value for each sample series. (**d**) Typical stress-strain diagram of a filler-free and crosslinked foam, which still contains the polyurethane template and of a filler-free foam pyrolyzed at 1000 °C.

The results are shown in Figure 11a. The characteristic compressive strength decreases up to a pyrolysis temperature of 400 °C and increases with rising pyrolysis temperatures. The compressive strength correlates with the total porosity of the foams. The higher the porosity, the lower is the compressive strength (see Figure 11a). The samples with SiC as filler material have higher compressive strength values in comparison to the Al_2_O_3_ filled foams. The Weibull moduli (Figure 11b) show a similar trend; with increasing compressive strength, the Weibull moduli increase. The values range between 3 and 11, lying in the range of as reported replica ceramic foams [49,50,51]. 

Due to the polymer-to-ceramic transformation the properties of the strut material change in density (Figure 3) and also the mechanical properties change [9]. The results of the fitted curves according to Equation (3) are shown in Figure 11c. Results of the correlated bending strength are given in the diagram and the values are increasing with increasing pyrolysis temperature. 

Using a fit with Equation (4), E was determined as the slope of the linear-elastic region in the stress-strain diagram. Exact numerical values are not shown here as many influencing factors related to the measuring system are present and therefore the numerical values are not meaningful. But there is a clear trend: As the pyrolysis temperature increases, the modulus of elasticity of the foams increases. This means that the foams lose elasticity and become more brittle due to ceramization. The highest increase in the modulus of elasticity was observed between 600 °C and 800 °C. Figure 11d represents a characteristic stress-strain diagram of a crosslinked and of a pyrolyzed polymer derived foam without fillers. The curves show the three typical regions of a foam under a compression load: the first linear-elastic region due to bending of the cell walls, the plateau region with almost constant stress and the third is the region of densification (not shown here) [52]. As an example, the crosslinked foams (130 °C) without fillers show a ductile behavior, due to the ductile PU-template that is still present in the crosslinked foam and the ductile polymer coating. The foams pyrolyzed at 1000 °C show a brittle behavior with a fluctuating stress-strain curve at the plateau region due to fractures of single struts. After reaching the maximal strength (crushing strength) these foams break catastrophically and the stress decreases rapidly. The foam samples pyrolyzed at 300 °C show a combination of both curves, and foams pyrolyzed at higher temperatures all show brittle behavior. 

## 4. Conclusions

Open macrocellular polymer derived ceramic foams were prepared via the replica technique and the changes in properties during the polymer-to-ceramic transformation have been demonstrated. Therefore, 20 ppi polyurethane foams were impregnated with a filler-free preceramic slurry or with slurries containing two different fillers, respectively. Thermal treatment was carried out in argon atmosphere between 130 °C (crosslinking) and 1000 °C (pyrolysis) and the changes in properties were studied in dependence of the pyrolysis temperature. In the temperature range from 400 °C to 600 °C main changes of foam properties occurred indicating the organic-to-inorganic transformation and the polyurethane decomposition. The mechanical behavior changes from elastic with the as crosslinked samples and those pyrolyzed at very low temperature to brittle, when pyrolysis was carried out at higher temperatures. The compressive strength increases with increasing pyrolysis temperature, due to densification and ceramization of the struts, but has a minimum at 400 °C, due to the beginning of the cleavage of the organic groups and thus a higher porosity. Carbon residues from decomposition of the polyurethane remaining in the hollow struts and deposit on the foams surface are detectable with SEM and Raman analysis. Due to the loss of the non-polar groups the surface wettability changes from hydrophobic in the crosslinked stage to hydrophilic in the pyrolyzed state. In summary, it was shown that the properties of polymer-derived foams change in a wide range from polymeric to ceramic, and thus, foams can be produced for a wide variety of applications.

## Figures and Tables

**Table 1:** Preceramic slurry compositions for the preparation of open porous polymer-derived-ceramics by the replication process with and without fillers. Sample designation oFS means filler-free samples.

Components	Filler Free (oFS)		Filled with Al_2_O_3_		Filled with SiC	
	wt%	vol%	wt%	vol%	wt%	vol%
**MTES**	4.23	5.20	7.95	14.34	8.08	14.03
**SILRES® H62 C**	38.06	37.08	22.94	32.77	22.63	31.12
**SILRES® MK**	57.09	57.14	22.94	33.66	22.63	31.97
**Filler**	–	–	45.88	18.80	46.34	22.44
**Aluminum acetylacetonate**	0.57	0.52	0.23	0.31	0.22	0.28
**Oleic acid**	0.04	0.05	0.07	0.13	0.09	0.16
